# Supplementary material for: A Humanized Yeast Phenomic Model of Deoxycytidine Kinase to Predict Genetic Buffering of Nucleoside Analog Cytotoxicity
Source: Genes (Basel). 2019 Sep 30;10(10):770. doi: 10.3390/genes10100770 (PMC6826991; doi:10.3390/genes10100770)
Supplement: Supplementary file 1 [file genes-10-00770-s001.zip › Additional_Files/Additional_File7_GOTermHeatmaps.zip!/Additional_File7_GOTermHeatmaps/meiosis_I.pdf]

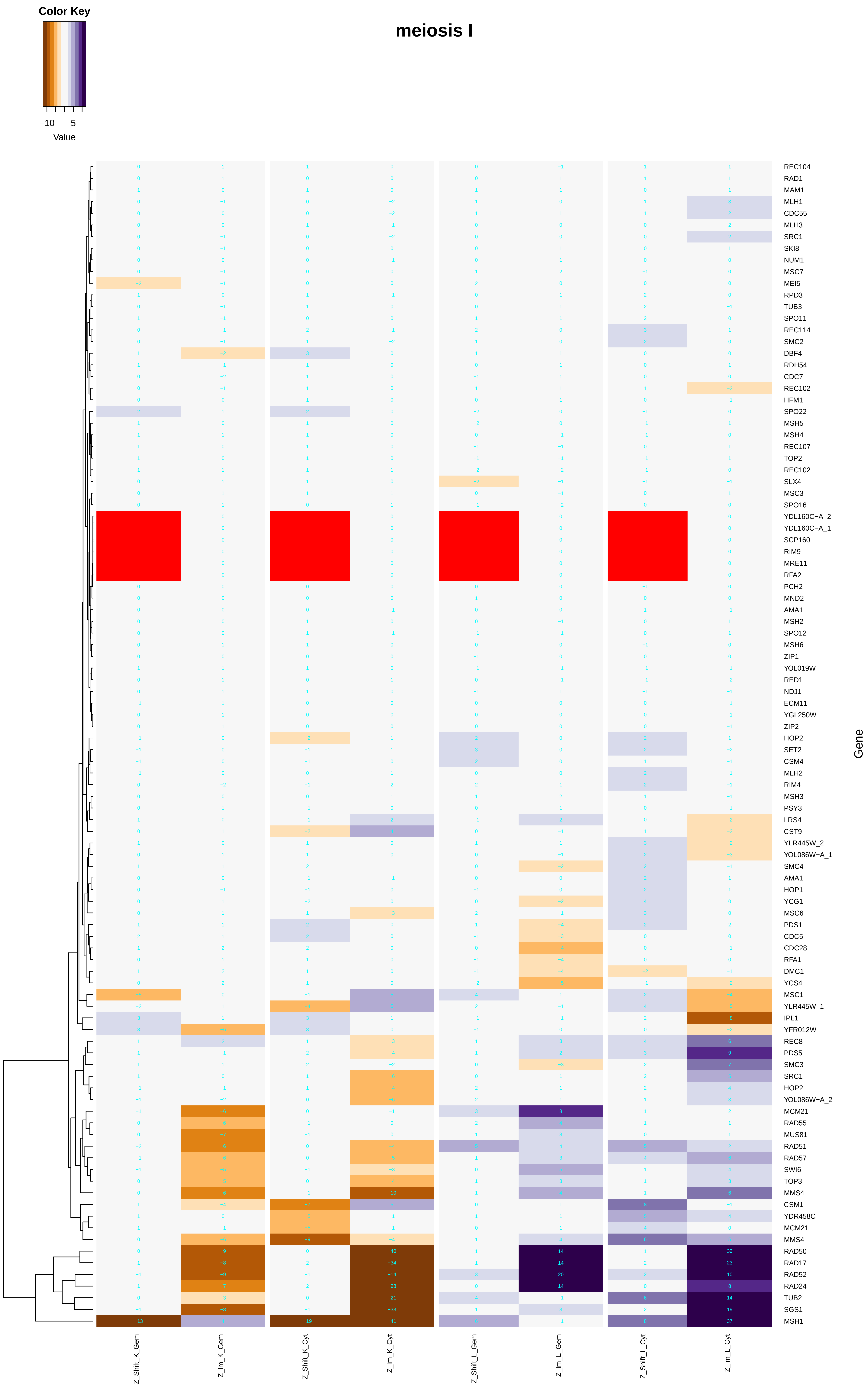

meiosis I
Color Key
−10
5
Value
Gene
REC104
RAD1
MAM1
MLH1
CDC55
MLH3
SRC1
SKI8
NUM1
MSC7
MEI5
RPD3
TUB3
SPO11
REC114
SMC2
DBF4
RDH54
CDC7
REC102
HFM1
SPO22
MSH5
MSH4
REC107
TOP2
REC102
SLX4
MSC3
SPO16
YDL160C−A_2
YDL160C−A_1
SCP160
RIM9
MRE11
RFA2
PCH2
MND2
AMA1
MSH2
SPO12
MSH6
ZIP1
YOL019W
RED1
NDJ1
ECM11
YGL250W
ZIP2
HOP2
SET2
CSM4
MLH2
RIM4
MSH3
PSY3
LRS4
CST9
YLR445W_2
YOL086W−A_1
SMC4
AMA1
HOP1
YCG1
MSC6
PDS1
CDC5
CDC28
RFA1
DMC1
YCS4
MSC1
YLR445W_1
IPL1
YFR012W
REC8
PDS5
SMC3
SRC1
HOP2
YOL086W−A_2
MCM21
RAD55
MUS81
RAD51
RAD57
SWI6
TOP3
MMS4
CSM1
YDR458C
MCM21
MMS4
RAD50
RAD17
RAD52
RAD24
TUB2
SGS1
MSH1
Z_Shift_K_Gem
Z_Im_K_Gem
Z_Shift_K_Cyt
Z_Im_K_Cyt
Z_Shift_L_Gem
Z_Im_L_Gem
Z_Shift_L_Cyt
Z_Im_L_Cyt
